# Electrically tunable third-harmonic generation using intersubband polaritonic metasurfaces

**DOI:** 10.1038/s41377-024-01517-y

**Published:** 2024-07-17

**Authors:** Seongjin Park, Jaeyeon Yu, Gerhard Boehm, Mikhail A. Belkin, Jongwon Lee

**Affiliations:** 1 Department of Electrical Engineering, Ulsan National Institute of Science and Technology (UNIST), Ulsan, Republic of Korea; 2 Walter Schottky Institute, Technical University of Munich, Garching, Germany

**Keywords:** Metamaterials, Nonlinear optics, Polaritons

## Abstract

Nonlinear intersubband polaritonic metasurfaces, which integrate giant nonlinear responses derived from intersubband transitions of multiple quantum wells (MQWs) with plasmonic nanoresonators, not only facilitate efficient frequency conversion at pump intensities on the order of few tens of kW cm^-2^ but also enable electrical modulation of nonlinear responses at the individual meta-atom level and dynamic beam manipulation. The electrical modulation characteristics of the magnitude and phase of the nonlinear optical response are realized through Stark tuning of the resonant intersubband nonlinearity. In this study, we report, for the first time, experimental implementations of electrical modulation characteristics of mid-infrared third-harmonic generation (THG) using an intersubband polaritonic metasurface based on MQW with electrically tunable third-order nonlinear response. Experimentally, we achieved a 450% modulation depth of the THG signal, 86% suppression of zero-order THG diffraction tuning based on local phase tuning exceeding 180 degrees, and THG beam steering using phase gradients. Our work proposes a new route for electrically tunable flat nonlinear optical elements with versatile functionalities.

## Abstract (web summary)

Electrically tunable mid-infrared third-harmonic generation is experimentally demonstrated using an intersubband polaritonic metasurface based on a multiple quantum-well structure with Stark tuning of the third-order nonlinear response.

## Graphical abstract

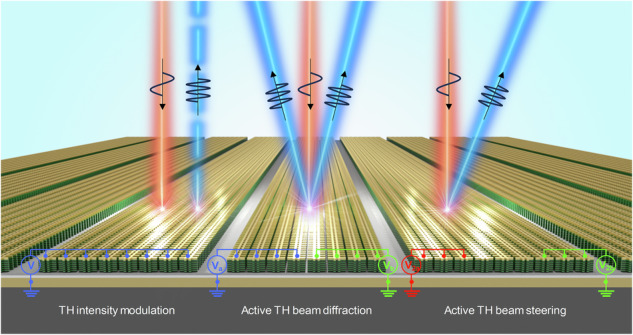

## Introduction

Optical metasurfaces, which are two-dimensional arrays of engineered subwavelength structures, have emerged as a transformative technology in the field of optics. They provide precise control over local scattering amplitude, phase, and polarization, offering unprecedented opportunities to manipulate optical signals at the deep subwavelength scale^1^. Beyond linear optics, nonlinear optical metasurfaces producing nonlinear optical processes, such as second- or third-harmonic generation (SHG or THG, respectively), in subwavelength-thin films are revealing new possibilities for flat nonlinear optics^2,3^. Their notable features, including relaxed phase-matching constraints, efficient frequency conversion, and precise control over nonlinear optical responses at deep subwavelength scales, showcase new avenues in this field. Numerous innovative applications are being explored based on nonlinear metasurfaces, including the generation of new frequencies for light sources^2,3^, nonlinear holography^4–9^, optical encryption^10–12^, nonlinear optical switching and modulation^13–17^, and quantum optical sources^18–21^. Implementing reconfigurable characteristics driven by external stimuli can significantly expand the utility of both linear and nonlinear metasurfaces^22^. This enables the implementation of multifunctionalities within a single device, diversification of operational wavelengths, and the development of applications based on dynamic beam manipulation.

Recently, various forms of nonlinear metasurfaces utilizing dielectric or metallic structures for efficient optical conversion have been investigated^2,3^. However, when employing natural nonlinear optical materials, the high light intensities and ultra-short laser pulses are required due to their low intrinsic nonlinear response. Inducing significant modulation of this intrinsic nonlinear response is also highly limited. Several investigations have shown electrical modulation of the nonlinear response amplitude using plasmonic or dielectric metasurfaces, relying on electric-field-induced SHG or optical rectification^23–26^. Recently, it has been reported that broadband third-order nonlinear susceptibility tuning and modulation of THG signals can be achieved through the electrical control of the Fermi energy level in graphene^27,28^. However, there is no reported information on the local phase tuning of the nonlinear response in these research findings.

The ultimate control of nonlinear optical responses is achieved when not only the magnitude but also the phase of nonlinear optical responses can be controlled at the meta-atom individual level. The first implementation of such characteristics was achieved in the intersubband nonlinear polaritonic metasurfaces^15^. The nonlinear polaritonic metasurfaces are formed through strong coupling between the giant nonlinear responses of intersubband transitions in the electron subbands within the conduction band of n-doped multiple quantum wells (MQWs) and electromagnetic cavity modes induced by plasmonic or dielectric resonators^15,29–35^. These metasurfaces have been studied for efficient frequency mixings, notably achieving a conversion efficiency of over 0.2% for SHG at subwavelength thickness^15,34^. The intersubband nonlinear response exhibits resonant characteristics that strengthen when the pump frequency approaches the intersubband transition^36,37^. By leveraging the intersubband Stark tuning effect, an electrically tunable nonlinear polaritonic metasurface has been implemented for SHG, enabling simultaneous tuning of the spectral, magnitude, and phase of the nonlinear response based on the applied voltage^15^. The intersubband nonlinear response modulated over a wide range depending on the applied voltage can induce efficient SHG over a broad spectrum when combined with arrays of meta-atoms of various sizes^38^. However, the electrically tunable nonlinear polaritonic metasurface has not been applied to other nonlinear processes, and the maximum phase tuning of the harmonic signal has been limited to 135°, allowing only limited dynamic beam manipulation.

**Fig. 1:**
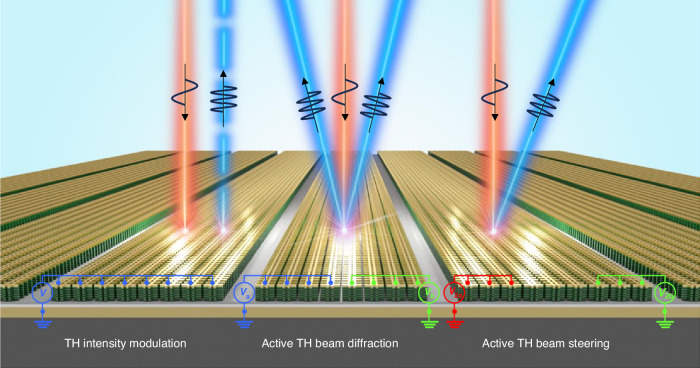
Electrically tunable nonlinear polaritonic metasurface for THG. Conceptual illustration of an electrically tunable nonlinear polaritonic metasurface and its use for third harmonic (TH) intensity modulation (left), dynamic modulation of TH beam diffraction (center), and dynamic TH beam steering (right) as reported in this paper

Here, we present, for the first time, nonlinear polaritonic metasurfaces for electrically tunable THG utilizing Stark tuning of the third-order nonlinear response of the MQW structure. We induced a giant third-order nonlinear response through resonant intersubband transitions between four spatially separated electron subbands in a structure of three-coupled quantum wells. The triply resonant intersubband nonlinearities allow for a far greater local phase tuning effect compared to the previous study^15^. To experimentally implement and leverage these characteristics, we demonstrated electrical intensity modulation of THG and dynamic beam manipulations using electrically induced phase grating and phase gradient structures, as illustrated in Fig. 1. Specifically, in the electrical diffraction modulation, we experimentally implemented 86% suppression of the zeroth-order beam occurring when achieving local phase tuning of over 180°.

## Results

### Design of multiple quantum-well structure

**Fig. 2:**
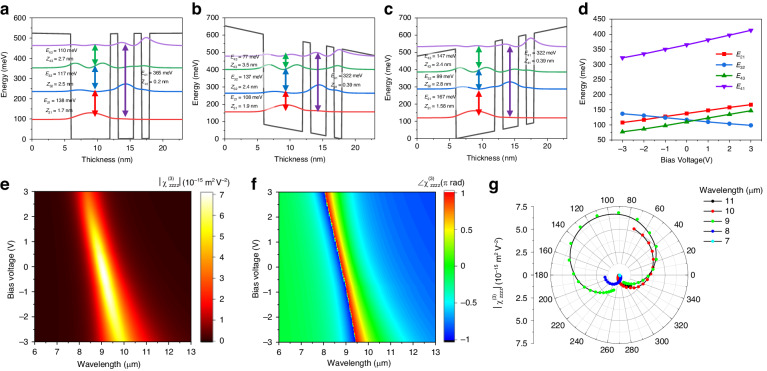
Intersubband nonlinearities of the MQW. **a**–**c** Conduction band diagram for a unit period of an In_0.53_Ga_0.47_As/Al_0.48_In_0.52_As coupled three-quantum-well structure designed for THG under the bias voltages of (**a**) 0 V, (**b**) −3 V, and (**c**) +3 V. *E*_*ij*_ and *eZ*_*ij*_ are the IST energy and transition dipole element, respectively, between electron subbands *i* and *j*. **d** Calculated IST energies over a bias voltage range from −3 V to 3 V with a step of 0.5 V. **e**, **f** Calculated magnitude (**e**) and phase (**f**) of the third-order intersubband nonlinear susceptibility of the MQW structure, ${\chi }_{zzzz}^{(3)}(V)$, as a function of wavelengths and applied bias voltage *V*. **g** Polar plot of the calculated magnitude and phase of the ${\chi }_{zzzz}^{(3)}(V)$ for an applied bias voltage range from −3 V to 3 V. A step of 0.2 V is added point by point starting from −3 V in a counterclockwise direction

For the proof-of-concept demonstration, we first designed a three-coupled-quantum-well unit structure using an In_0.53_Ga_0.47_As/Al_0.48_In_0.52_As heterostructure as shown in Fig. 2a. The layer sequence of the unit structure is **6**/6/**1.2**/2.4/**1.2**/1.2/**6** nm, where boldface indicates Al_0.48_In_0.52_As barriers and the first 6 nm well layer is n-doped with a density of 1.5 × 10^18^ cm^−3^. A total 400 nm thick MQW layer optimized for efficient THG is constructed by repeating the unit period 17 times. In this configuration, giant third-order nonlinear susceptibility,${\chi }_{zzzz}^{(3)}$, for the surface normal direction (z-direction here) is induced by resonant transitions between the first four electron subbands. Figure 2b, c show the conduction band diagram of the unit structure under applied bias voltages of −3 V and +3 V, respectively, ensuring the safe operation of the device. Given the spatially separated electron subbands, the intersubband transition energies, *E*_*ij*_ between electron subbands *i* and *j*, can be modulated by the applied bias voltage to the MQW layer due to the quantum-confined Stark effect (QCSE) of the intersubband transitions. The intersubband transition energies according to the bias voltage ranging from −3 V to +3 V is shown in Fig. 2d. The designed MQW structure was grown using molecular-beam epitaxy and experimentally measured IST energies were well matched with the calculation results (see Supplementary Information).

The electrical modulation of ISTs leads to the electrically tunable third-order nonlinear susceptibility, ${\chi }_{zzzz}^{(3)}(V)$. Considering resonant transitions between the first four electron subbands in the MQW structure, ${\chi }_{zzzz}^{(3)}(V)$ can be expressed as functions of a pump wavelength and an applied bias voltage to the MQW layer^36^:

1 $${\chi }_{zzzz}^{(3)}(\omega \to 3\omega ,\,V)\approx \frac{{N}_{e}{e}^{4}}{{\varepsilon }_{0}}\frac{{Z}_{12}(V){Z}_{23}(V){Z}_{34}(V){Z}_{41}(V)}{(\hslash \omega -{E}_{12}(V)-i\hslash {\gamma }_{12})(2\hslash \omega -{E}_{13}(V)-i\hslash {\gamma }_{13})(3\hslash \omega -{E}_{14}(V)-i\hslash {\gamma }_{14})}$$

where *N*_*e*_ is the averaged electron density, *e* is the electron charge, *ω* is the pump frequency, $\hslash$ is the reduced Planck constant, *E*_*ij*_(*V*) and *eZ*_*ij*_(*V*) are the IST energy and dipole moment as a function of bias voltage *V*, and $\hslash {\gamma }_{ij}$ is the half-width at half-maximum linewidth of the IST between electron subband *i* and *j*. The calculated magnitude and phase spectra of the ${\chi }_{zzzz}^{(3)}(V)$ for the bias voltage ranging from −3 V to +3 V are shown in Fig. 2e, f, respectively. The MQW structure has a peak $|{\chi }_{zzzz}^{(3)}|$ value of 7.15 × 10^−14^ m^2^ V^−2^ at a pump wavelength of 9μm. The IST energies, modulated to different magnitudes by the QCSE, tune the peak wavelength of ${\chi }_{zzzz}^{(3)}(V)$ depending on the bias voltage. Specifically, a positive bias induces a blueshift, while a negative bias leads to a redshift, of the maximum magnitude of the THG response demonstrating broad spectral tunability spanning 8.4 to 10 μm in response to the bias voltage range of −3 V to +3 V. Additionally, a significant phase modulation of up to 1.7π radians is achievable at the 9 μm wavelength with the same range of applied bias voltages. However, in this case, the magnitude of ${\chi }_{zzzz}^{(3)}(V)$ will also vary with applied voltage. For a better illustration of the complex nonlinear susceptibility element, Fig. 2g depicts its magnitude and phase in a polar plot, corresponding to a voltage range of −3 V to +3 V at five different wavelengths. Our ability to modulate the magnitude and phase of the intersubband ${\chi }_{zzzz}^{(3)}(V)$ in a MQW can be combined with a suitable plasmonic nanocavity to construct a reconfigurable nonlinear metasurface. This allows for electrical control over the spectral, magnitude, and phase characteristics of THG at the individual meta-atom level.

### Design of metasurfaces for THG

**Fig. 3:**
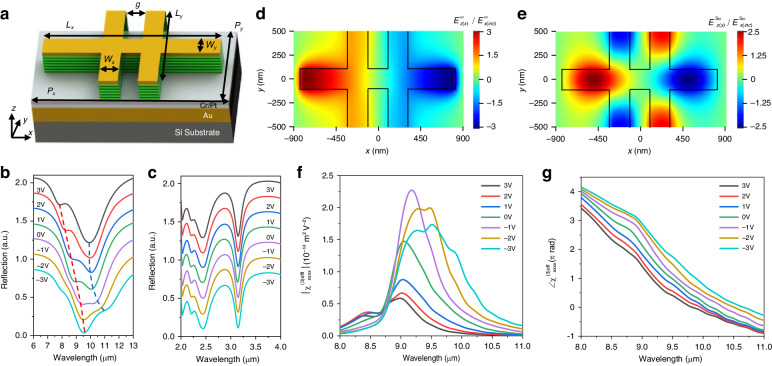
Numerical simulations of the metasurface. **a** Schematic of the meta-atom unit structure. The dimensions are *L*_x_ = 1.6 μm, *L*_y_ = 1 μm, *w*_x_ = *w*_y_ = *g* = 200 nm, *P*_x_ = 1.8 μm, and *P*_y_ = 1 μm. **b**, **c** Simulated reflection spectra of the metasurface over an applied bias voltage range from −3 V to +3 V at a resolution of 1 V under x-polarized incident light in the (**b**) FF range and (**c**) TH frequency range. For better illustration, the reflection spectra at each bias voltage are vertically separated by 0.2. **d**, **e** Top-view cross section of the normalized E_z_ field enhancement distribution in the MQW layer, located 100 nm beneath the top MQW surface at the (**d**) FF and (**e**) TH frequencies. **f**, **g** Calculated magnitude (**f**) and phase (**g**) spectra of the effective third-order nonlinear susceptibility of the metasurface,${\chi }_{xxxx}^{(3)eff}(V)$, as a function of the pump wavelength over an applied bias voltage range from −3 V to +3 V at a resolution of 1 V

For efficient THG and the application of bias voltages to the MQW layer, we designed a meta-atom unit structure by sandwiching the MQW layer between an optically thick bottom metal layer and a top plasmonic nanoresonator, as shown in Fig. 3a. The plasmonic nanoresonator was configured in the shape of an intersection of a rod bar along the x-axis and two load lines along the y-axis. These load lines are connecting neighboring meta-atoms in the y-direction for biasing. The elements of the effective third-order nonlinear susceptibility tensor of the metasurface are expressed as shown below^32^,

2 $${\chi }_{ijkl}^{(3)eff}(V)={\chi }_{zzzz}^{(3)}(V){\int }_{{V}_{MQW}}\left(\frac{{E}_{z(i)}^{3\omega }(x,y,z)}{{E}_{i(inc)}^{3\omega }}\cdot \frac{{E}_{z(j)}^{\omega }(x,y,z)}{{E}_{j(inc)}^{\omega }}\cdot \frac{{E}_{z(k)}^{\omega }(x,y,z)}{{E}_{k(inc)}^{\omega }}\cdot \frac{{E}_{z(l)}^{\omega }(x,y,z)}{{E}_{l(inc)}^{\omega }}\right)dV/{V}_{unit}$$

where ${E}_{z(i)}^{\omega }$ and ${E}_{z(i)}^{3\omega }$ are the local E_z_ field components in the MQW region at frequencies of ω and 3ω, respectively, induced by *i*-polarized incident waves ${E}_{i(inc)}^{\omega }$ and ${E}_{i(inc)}^{3\omega }$, respectively, *V*_*unit*_ and *V*_*MQW*_ are volumes of the MQW region before and after etching, respectively. The meta-atom structure was designed to exhibit plasmonic resonances under x-polarized incident light at both the fundamental frequency (FF) ω and the third harmonic (TH) frequency 3ω. In this configuration, the element of the effective nonlinear susceptibility tensor for the xxxx polarization, ${\chi }_{xxxx}^{(3)eff}$, will be dominant, with the first letter referring to the TH polarization and the last three letters indicating the FF input pump polarization. The other tensor elements have negligible values compared to ${\chi }_{xxxx}^{(3)eff}$. Figures 3b, c show the simulation results of reflection spectra under various bias voltages for normal incidence of x-polarized light. Strong absorption peaks due to plasmonic resonances are observed near the 9 μm pump wavelength and 3 μm TH wavelength. Especially near 9 μm, the presence of polaritonic peak splitting is noticeable due to the strong coupling of the IST and the cavity mode. The wavelengths of these two peaks undergo shifts in response to the applied bias voltage, confirming the Stark tuning of the IST. The strong absorption near the TH frequency ensures out-coupling of the TH signal generated in the MQW region to free space through reciprocity^39^, and no spectral tuning was observed with respect to the applied bias voltage for this resonance. Figure 3d, e show the simulation results of the E_z_ field enhancement distribution, ${E}_{z(x)}^{\omega \,or\,3\omega }/{E}_{x(inc)}^{\omega \,or\,3\omega }$, monitored in the MQW region for the x-polarized normal incident waves at the FF ω and TH frequency 3ω, respectively. Figure 3f, g show the calculated magnitude and phase spectra of ${\chi }_{xxxx}^{(3)eff}$ for the bias voltage ranging from −3 V to +3 V with a step of 1 V. The maximum value of $|{\chi }_{xxxx}^{(3)eff}|$ is 2.3 × 10^−14^ m^2^ V^−2^ under the bias voltage of −1 V, and spectral peak tuning from 9 to 9.5 μm was achieved within the −3 V to +3 V bias voltage range. The phase response of ${\chi }_{xxxx}^{(3)eff}$ was calculated across the bias voltage range, revealing a uniform tuning of the phase in the 8–11 μm wavelength range. Particularly, at a wavelength of 9 μm, a local phase tuning of 1.5π radians was observed.

### Experiments

**Fig. 4:**
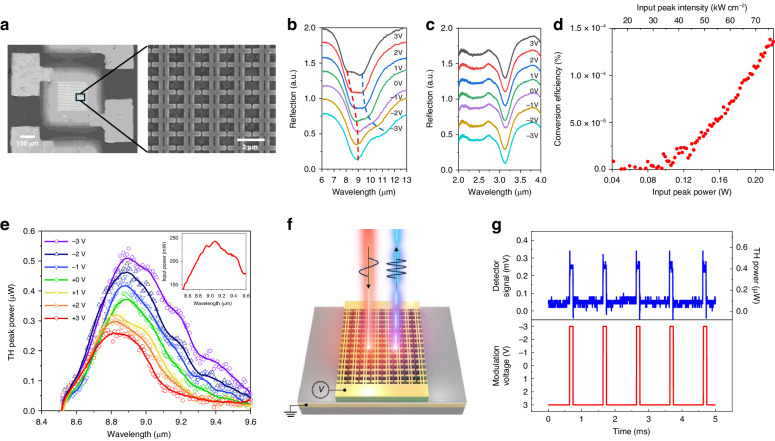
Linear and nonlinear optical characterization of the metasurface. **a** SEM images of the fabricated metasurface with electrical contact pads and enlarged image to show the meta-atom arrays. **b**, **c** Measured reflection spectra of the metasurface over an applied bias voltage range from −3 V to 3 V at a resolution 1 V under x-polarized incident light in the (**b**) FF range and (**c**) TH frequency range. **d** Measured TH power conversion efficiency as a function of the peak input pump power (bottom x-axis) or input intensity (top x-axis). **e** Measured TH power spectra (circle: measured data, line: moving average) as a function of the pump wavelength for different bias voltages. Inset shows the input pump power spectra. **f** Schematic image of the TH intensity modulation from the metasurface. **g** Dynamic TH intensity modulation at a pump wavelength of 9.1 µm. Top and bottom panels are the time dependence of the InSb detector signal and the corresponding TH power change (top panel) for the applied square-modulated bias voltage between −3 V and +3 V with a 10% duty cycle at a 1 kHz frequency (bottom panel)

To experimentally validate our concept, we fabricated metasurfaces with dimensions of 200 μm × 200 μm, as depicted in the scanning electron microscopy (SEM) image in Fig. 4a. The linear reflection spectra of the fabricated metasurfaces under the DC bias voltages varying from −3 V to +3 V were measured using a Fourier transform infrared spectrometer equipped with an infrared microscope as shown in Fig. 4b, c. Polaritonic peak splitting and spectral tuning were observed around the FF for the applied bias voltage range, indicating strong coupling and Stark tuning of the IST. This experimental observation is in good agreement with the simulation results (cf. Fig. 3b, c).

For the nonlinear optical characterization of the fabricated metasurface, we established an experimental setup utilizing a wavelength-tunable quantum cascade laser (QCL) as a pump source (the optical setup is shown in the Supplementary Fig. S3). The maximum TH power conversion efficiency of 1.38 × 10^−4^% at 0 V was achieved at a pump intensity of 77 kW cm^-2^ and a pump wavelength of 8.9 μm as shown in Fig. 4d. Figure 4e illustrates the measured TH peak power spectra as a function of the pump wavelength for different bias voltages, with the pump power spectrum represented in the inset. The spectral position of the maximal THG was slightly tuned from 8.82 μm to 8.9 μm of the pump wavelength by changing the bias voltage from −3 V to +3 V, resulting from the Stark-tuning of intersubband nonlinearity. However, the measured results of spectral tuning are notably smaller than the simulated results. This discrepancy is attributed to nonuniform conduction band bending arising from the formation of Schottky contacts at the interfaces between the MQW and metallic contact layers (see Section 2 in Supplementary Information). The spectral tuning of third-order nonlinear response of the metasurface enables electrical modulation of TH intensity at a fixed wavelength as illustrated in Fig. 4f. As shown in Fig. 4g, we experimentally achieved a 450% modulation depth in the THG signal at the pump wavelength of 9.1 μm by applying a square-modulated bias voltage between −3 V and +3 V with a frequency of 1 kHz. The modulation depth is defined as $[{P}_{TH,MAX}(-3V)-{P}_{TH,\min }(3V)]/{P}_{TH,\min }(3V)$ where ${P}_{TH,MAX}({P}_{TH,\min })$ is the maximum (or minimum) TH power. The calculated resistance-capacitance (RC) time constant, taking into account the device dimensions within the modulated voltage range, was 47.1 ns, which corresponds to a cutoff THG modulation frequency of 3.38 MHz (see Supplementary Information).

**Fig. 5:**
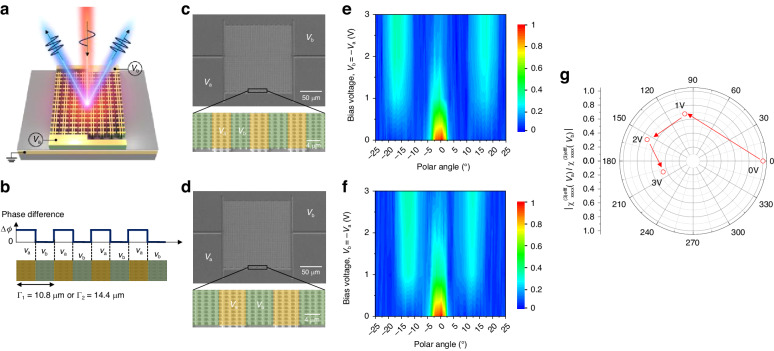
Dynamic TH beam diffraction control. **a** Schematic image of the TH beam diffraction from the electrically induced phase grating metasurface. **b** Spatial phase profile of the phase grating metasurfaces for supercell periods of Γ_1_ = 10.8 μm or Γ_2_ = 14.4 μm. The phase of the section connected to *V*_b_ is the reference. **c**, **d** SEM images of the fabricated metasurfaces for supercell periods of Γ_1_ (**c**) and Γ_2_ (**d**). Bottom panels show zoom-in view of the meta-atom arrays and the distributions of applied bias voltages. **e**, **f** Measured far-field profiles of the generated TH signal as a function of polar angle and applied bias voltage from 0 V to +3 V for supercell periods of Γ_1_ (**e**) and Γ_2_ (**f**). **g** Complex value ratio of the two extracted effective third-order nonlinear susceptibilities for the two bias voltages *V*_a_ and *V*_b_, ${\chi }_{xxxx}^{(3)eff}({V}_{a})/{\chi }_{xxxx}^{(3)eff}({V}_{b})$

In addition to the dynamic TH intensity modulation, local phase tuning of the nonlinear response for each individual unit of the meta-atom enables dynamic wavefront manipulation of the THG. As an application of this unique feature of our device, we conducted an experiment involving an electrically tunable phase grating metasurface at the pump wavelength of 8.9 μm to produce TH diffraction beam tuning, as illustrated in Fig. 5a. In this experiment, six and eight rows of meta-atoms forming supercell periods of Γ₁ = 10.8 μm and Γ₂ = 14.4 μm were used and two bias voltages *V*_a_ and *V*_b_, were applied to the two repeating subsections to form an electrically induced phase grating with a 50% duty cycle (Fig. 5b). The fabricated metasurfaces are shown in Fig. 5c, d. By repeating the supercell in the lateral direction, the phase grating metasurfaces were constructed as shown in the bottom panels of Fig. 5c, d. According to the diffraction grating equation ($\sin \theta =\pm m{\lambda }_{TH}/\varGamma$), the first order TH diffraction angles are calculated to be ±16.1 and ±12 degrees for the supercell periods of Γ₁ and Γ₂, respectively. Figure 5e, f present the measured TH intensity profiles as a function of deflection angle relative to surface normal for the applied bias voltages in the form of *V*_a_ = −*V*_b_ for the two metasurfaces. The TH intensity was normalized to the peak THG signal at *V*_a_ = *V*_b_ = 0 V. When *V*_a_ = *V*_b_ = 0 V, THG occurs in the surface-normal direction due to the lack of a phase difference between the metasurface sections. With an increase in the magnitude of the bias voltage, the first order TH diffraction signal increases, while the zeroth order signal is gradually suppressed. Upon applying *V*_a_ = −3 V and *V*_b_ = 3 V, the induced spatial phase difference leads to the generation of ±1 diffraction orders, accompanied by the 86% suppression of the zeroth-order TH signal. We extracted the phase change according to the bias voltage by comparing the measurement results with the simulation for the far-field profiles of the phase grating metasurface (Supplementary Figs. S6 and S7). Figure 5g presents the ratio of the two complex nonlinear susceptibilities, ${\chi }_{xxxx}^{(3)eff}({V}_{a})/{\chi }_{xxxx}^{(3)eff}({V}_{b})$, in a polar plot. The magnitude of the ratio of the two nonlinear susceptibilities gradually decreases from 1 to 0.2 and the phase of the ratio increases from 0 to 1.1π radians, as the magnitude of the bias voltage was increased from 0 to 3 V.

**Fig. 6:**
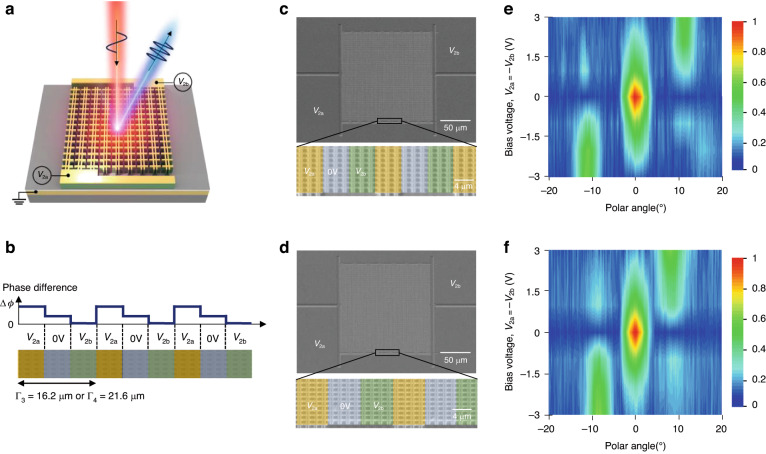
Dynamic TH beam steering measurements. **a** Schematic image of the TH beam steering from the electrically induced phase gradient metasurface. **b** Spatial phase profile of the phase gradient metasurfaces for supercell periods of Γ_3_ = 16.2 μm and Γ_4_ = 21.6 μm. The phase of section connected to *V*_b_ is the reference. **c**, **d** SEM image of the metasurface for supercell periods of Γ_3_ (**c**) and Γ_4_ (**d**). Bottom panels show zoom-in view of the meta-atom arrays and the distribution of applied bias voltages. **e**, **f** Measured far-field profiles of the generated TH signal as a function of polar angle and applied bias voltage from 0 V to +3 V for supercell periods of Γ_3_ (**e**) and Γ_4_ (**f**)

Moreover, dynamic beam steering for THG was demonstrated using electrically tunable phase gradient metasurfaces as illustrated in Fig. 6a. The metasurface is constructed by repetitively arranging supercells with three phase sections in the lateral direction. Each phase section includes three or four rows of meta-atoms forming supercell periods of Γ_3_ = 16.2 μm or Γ_4_ = 21.6 μm, respectively. Voltage biases, *V*_2a_ and *V*_2b_ are applied to the left and right phase sections, respectively, while no voltage is applied to the central section (Fig. 6b). SEM images of the fabricated metasurfaces are shown in Fig. 6c, d. The measured TH intensity profiles as a function of the bias voltage are shown in Fig. 6e, f. A higher bias voltage induces a larger phase difference, leading to an increased intensity of the steered TH signal and the suppression of the TH beam in the normal direction. For *V*_2a_ = *V*_2b_ = 0 V, THG occurs in the surface normal direction due to the absence of the phase difference between the sections. However, with *V*_2a_ = −*V*_2b_ = 3 V, the TH beam is generated at +10.7 and +8 degrees for the supercell periods of Γ_3_ and Γ_4_, respectively, in accordance with the generalized Snell’s law^40^. Reversing the sign of the bias voltage results in TH beams generated in the opposite direction at −10.7 and −8 degrees.

## Discussion

In conclusion, we reported a novel approach to produce electrical beam steering in THG nonlinear metasurfaces. Our metasurfaces provide a giant third-order nonlinear response up to 7.15 × 10^−14^ m^2^ V^−2^ for 9 μm pump wavelength which enables us to experimentally obtain a THG power conversion efficiency of 1.38 × 10^−4^% at a pump intensity of only 77 kW cm^−2^. The THG power conversion efficiency can be enhanced when utilizing volume resonant modes induced by Mie resonance in an all-dielectric resonator structure^34^. We note that the flexibility inherent in the IST within MQWs allows one to adapt the metasurface operational wavelengths to specific requirements from near IR to THz. Through application of bias voltage that produced Stark tuning of ISTs, we achieved control over both amplitude and phase of the nonlinear optical response at the individual meta-atom level. With this feature of our device, we experimentally demonstrated THG spectral tuning, TH intensity modulation, as well as dynamic TH beam diffraction and steering. Our metasurface exhibited significant phase tuning of 1.1π radians of the THG nonlinear response within a modest applied bias voltage range of −3 to +3 V. This relatively wide range of phase tuning is attributed to the triply resonant electronic transitions in the MQW as shown in Eq. (1). Further increased phase tuning can be achieved by utilizing four spatially separated electron subbands induced by four coupled quantum wells, albeit with a sacrifice of THG conversion efficiency. Exploiting the THG spectral tuning capability, we achieved a TH intensity modulation depth of 450% at a wavelength of 9.1 μm. With the local phase tuning of over π radians, we demonstrated dynamic TH beam diffraction tuning and TH beam steering with 86% suppression of the zeroth order beam. Our achievements in electrically tunable nonlinear metasurfaces for THG pave the way for innovative applications requiring dynamic intensity modulation or dynamic wavefront manipulation.

## Materials and methods

### Numerical simulation

For the meta-atom simulation, we utilized a finite-difference time-domain solver (Lumerical FDTD) with periodic boundary conditions in the x- and y-directions and the perfect matched layer condition in the z-direction. The built-in dielectric constant of the Au layer in the FDTD software was used for the simulation. The out-of-plane and in-plane dielectric constants of the MQW layer (${\varepsilon }_{\perp }(\omega )$ and ${\varepsilon }_{\parallel }(\omega )$, respectively; see Supplementary Fig. S1) were modeled using the measured intersubband absorption data and the calculated transition dipole elements based on the Poisson-Schrödinger solver, and these values were incorporated into FDTD simulations. The IST energies under applied voltage were computed using the Poisson- Schrödinger solver, enabling the calculation of linear and nonlinear optical responses of the metasurface as a function of applied voltage, as illustrated in Fig. 3 and Supplementary Fig. S5.

### Device fabrication

Bottom metallic layers, consisting of a sequentially deposited 20 nm thick layer of chromium (Cr), a 50 nm thick layer of platinum (Pt), and a 150 nm thick layer of gold (Au), were applied to both the MQW layer and another silicon wafer. These two wafers underwent thermo-compressive bonding with the metallic layers facing each other, subject to a pressure of 1.5 kN cm^-^² and a temperature of 240 °C for 15 minutes. To expose the MQW layer, selective chemical wet etching techniques were employed. Initially, the InP substrate on the MQW layer side was removed using a solution composed of HCl and deionized (DI) water in a 3:1 ratio, aided by a 300 nm thick In_0.53_Ga_0.47_As etch-stop layer. Subsequently, the InGaAs etch-stop layer was selectively etched using a solution of Citric Acid and H_2_O_2_ in a 2:1 ratio, revealing the Al_0.48_In_0.52_As etch-stop layer. Finally, the 100 nm thick Al_0.48_In_0.52_As etch-stop layer was removed using a selective wet etching solution of HCl and DI water in a 3:1 ratio. Upon exposing the MQW layer, a 6 nm thick layer of titanium (Ti) and a 50 nm thick layer of Au were evaporated onto it, and a 470 nm thick silicon nitride (Si_3_N_4_) hard-mask layer was deposited via plasma-enhanced chemical vapor deposition (PECVD). Nanoresonator arrays were patterned onto the SiN mask layer through electron beam lithography, and these patterns were etched through the SiN layer using reactive ion plasma etching (RIE). The patterned SiN layer served as an etch mask to further etch through the 50 nm thick gold layer and the 400 nm thick MQW layer using inductively coupled plasma RIE. Subsequently, the SiN mask was removed using selective chemical wet etching in buffered oxide etchant (BOE) at a 6:1 ratio. To prevent current spreading, a mesa structure with dimensions of 400 µm × 400 µm was created, and a 450-nm-thick SiN passivation layer was deposited on the sample via PECVD. Following the creation of a pattern opening, a top contact layer of 20 nm Cr/300 nm Cu/20 nm Cr/50 nm Au was patterned. Finally, the device was attached to an aluminum plate using silver paste. The entire fabrication process is illustrated in Supplementary Fig. S2.

### Optical characterization

We measured the linear reflection spectra of the metasurfaces using a Fourier Transform Infrared (FTIR) spectrometer equipped with an IR microscope (Bruker, Vertex 70 and Hyperion 1000). For nonlinear optical characterization, a wavelength-tunable quantum cascade laser (QCL) in pulse-wave mode (Daylight Solutions, Mircat system) was employed with a tuning range of 909 – 1230 cm^-1^, peak power of 400 mW, and a repetition rate and duty cycle for pulse mode set at 100 kHz and 10%, respectively. A calibrated InSb photodetector (Electro Optical System, Inc., bandwidth: DC – 200 kHz) was utilized, as depicted in Supplementary Fig. S3. The focal spot diameter at the sample position, confirmed by the knife-edge measurement, was 2w = 27 μm. A Gaussian intensity profile was assumed for both the FF input pump beam (${I}_{FF}{e}^{-2{r}^{2}/{w}^{2}}$) and the TH beam (${I}_{TH}{e}^{-6{r}^{2}/{w}^{2}}$). The thermal power meter (Thorlabs, S302C) was utilized to measure the average pump power. DC bias voltage was applied through a source meter (Keithley, SMU 2450) and a DC power supply (HP, E3631A). For dynamic modulation of the TH signal, a square voltage pulse was applied using a high-voltage pulse generator (HP, 8114 A), with the detector signal being monitored through an oscilloscope (Tektronix, TDS 2024 C). In the nonlinear beam steering measurements employing the tunable phase grating and gradient metasurface, the InSb detector underwent lateral scanning without a ZnSe lens positioned in front. The lateral shift, denoted as *d*, of the signal was expressed as $d=f\,\tan \theta$, where *f* represents the effective focal length of the ZnSe focusing lens, and *θ* denotes the angle of the TH signal concerning the normal beam path.

### Supplementary information

Supplementary Information

## Data Availability

The data that support the plots within this paper and other findings of this study are available from the corresponding author upon reasonable request.
