# Supplementary material for: Electrically tunable third-harmonic generation using intersubband polaritonic metasurfaces
Source: Light Sci Appl. 2024 Jul 17;13:169. doi: 10.1038/s41377-024-01517-y (PMC11254926; doi:10.1038/s41377-024-01517-y)
Supplement: Supplementary file 1 — Supplementary Information [file 41377_2024_1517_MOESM1_ESM.docx]

Supplementary Information for

**Electrically tunable third-harmonic generation using intersubband polaritonic metasurfaces**

Seongjin Park^1^, Jaeyeon Yu^1^, Gerhard Boehm^2^, Mikhail A. Belkin^2^, and Jongwon Lee^1*^

^1^Department of Electrical Engineering, Ulsan National Institute of Science and Technology (UNIST), Ulsan 44919, Republic of Korea

*^2^Walter Schottky Institute, Technical University of Munich, Am Coulombwall 4, Garching 85748, Germany*

*^*^Corresponding author. E-mail:* [*jongwonlee@unist.ac.kr*](mailto:jongwonlee@unist.ac.kr)

**1. Intersubband absorption measurement**

The experimental setup for intersubband absorption measurement is represented in Figure S1a. Figure S1b shows experimentally measured absorbance spectrum of the MQW sample which is sandwiched between optically thick gold layers. Because the MQW absorbs only TM polarized light along the growth direction, the intersubband absorption coefficient can be determined by normalizing the TM polarized signal to TE polarized signal after passing the MQW. The intersubband absorption coefficient, α_W_, is expressed as: [Ref. S1]

$\alpha_{W}=-\frac{1}{L_{\mathrm{int}}}\ln(10)\log_{10}\left(\frac{I_{TM}}{I_{TE}}\right)$ (S1)

where $L_{\mathrm{int}}=n_{p}L_{MQW}/\cos45^{\circ}=5.656\text{ }\mu\text{m}$ is the MQW interaction length of the multipath sample piece, *I_TM_* and *I_TE_* are the measured signal for TM and TE polarization, respectively. Transition energies of $\hbar\omega_{12}= 137 meV$, $\hbar\omega_{14}=258 meV$ and $\hbar\omega_{14}= 375 meV$ were obtained which are corresponding to E_12_, E_13_ and E_14_, respectively (see Figure S1b). The transition linewidths are $2\hbar\gamma_{1}= 24 meV$, $2\hbar\gamma_{13}= 47 meV$ and $2\hbar\gamma_{14}=52 meV$, and the intersubband absorption coefficient, $\alpha_{W}$, are 5.32× 10^3^ cm^-1^, 8.81 × 10^2^ cm^-1^ and 5.07 × 10^2^ cm^-1^ for the 1-2, 1-3 and 1-4 transitions, respectively. From the intersubband absorption measurement, the imaginary part of the surface normal component of dielectric function can be determined using the equation expressed as a function of the absorption coefficient as:

$\mathrm{Im}\left(\sqrt{\varepsilon_{\perp}}\right)=\frac{\lambda}{4\pi}\alpha_{W}$ (S2)

From the equation, we extracted the imaginary part of $\varepsilon_{\perp}$ using the absorption coefficient spectra from the intersubband absorption measurement. In a different way, the surface normal component of dielectric function $\varepsilon_{\perp}$ of the MQW structure can be modeled as following equation:

(S3)

where $\varepsilon_{core}$is the averaged dielectric constant of the undoped semiconductor heterostructures, *N_e_* is the averaged doping density, *e* is the electron charge, *ω* is the pump frequency, , , and $ez_{ij}$are the transition energy, linewidth, and dipole moment, respectively, for the intersubband transitions between the electron subband *i* and *j*. We estimated the averaged electron density, *N_e_*, to calculate the third-order nonlinear susceptibility, $\chi_{zzzz}^{(3)}$ and the averaged dielectric constant to determine MQW dielectric constant in surface parallel direction which is expressed as:

$\varepsilon_{\parallel}(\omega)\approx\varepsilon_{core}(\omega)+i\frac{N_{e}e^{2}\tau_{D}}{\varepsilon_{0}\omega m^{*}\left(1-i\omega\tau_{D}\right)}$ (S4)

where $\tau_{D}\approx10^{-13}\text{ s}$is the Drude relaxation time and we assumed free electron motion in the plane of the semiconductor layer.

**2. Non-uniform conduction band bending effect**

The current-voltage characteristic of the fabricated metasurface is represented in Supplementary Figure S4a. The I-V curve implies Schottky contacts formation at both the upper and lower MQW-metal junctions. Considering this Schottky contact formation, we derived the conduction band diagram of the metasurface using Poisson-Schrodinger solver (Nextnano), illustrated in Supplementary Figures S4a-h for different bias voltages and, electric field strength spectra shown in Supplementary Figure S4i. Our observations indicate a non-uniform bending of the conduction band upon bias voltage application. With positive bias, there's prominent band bending in the MQW layer near the bottom metal contact, and this bending is less distinct in other regions. Conversely, under negative bias applications, there's significant band bending near the top metal contact and lesser bending at other regions. For the FDTD simulation we segmented our analysis into two sections based on the non-uniform conduction band bending (Supplementary Table S1). The simulated reflection spectra of the metasurface for x-polarized incident light is presented in Supplementary Figure S5a. Notably, the peak splitting is narrower compared to the original simulated reflection spectra in Figure 3b, aligning more closely with the measured reflection spectra. Supplementary Figures S5b and S5c show the calculated amplitude and phase of the nonlinear third-order susceptibility of the metasurface, having narrower peak tuning range from 8.7 µm to 9.48 µm and phase tuning of 1.25 π radian at a wavelength of 9 µm than original calculated results (Figure 3f and 3g).

**3. TH beam steering simulation**

In the FDTD simulation for TH beam manipulation, dipole antennas are positioned at each unit cell of the metasurface. In the case of THG phase grating metasurface, a total of 5 supercells were placed with the boundary set to periodic. The amplitude of each dipole antenna is determined based on the measured TH power spectrum, and its phase is adjusted according to the bias voltage to match the measured result. Phase changes according to the bias voltage were estimated by comparing the measurement results with the simulation for far-field profiles of the phase grating metasurface under a single bias application, varying V_a_ from -3 V to 1 V with a resolution of 1 V while V_b_ remains constant at 0 V. Measurement results are shown in Supplementary Figures S6a-g and simulation results are Supplementary Figures S7a-g. Resultant phases, normalized to the phase for a bias voltage at 0 V, are presented in Supplementary Figure S7h as a function of bias voltage.

**4. Cut-off frequency of the THG intensity modulation**

The THG intensity modulation results shown in Fig. 4g were measured over the voltage range from -3 V to +3 V, and the average resistance of the device obtained through I-V measurements within this range is *R_av_* = 600 Ω (see Supplementary Fig. S4a). The dimension of the mesa structure containing the MQW layer with 400 nm thickness (*L*_MQW_) is *A*_mesa_ = 400 μm × 400 μm and the dimension of the contact pad containing the SiN passivation layer with 320 nm thickness (*L*_SiN_) is *A*_contact_ = 2 × 300 μm × 200 μm. The averaged dielectric constant of the MQW and the SiN layer are $\varepsilon_{MQW}=13.25\varepsilon_{0}$ and $\varepsilon_{SiN}=9.5\varepsilon_{0}$. The capacitance of the structure can be calculated as $C_{meta}=\frac{\varepsilon_{MQW}A_{mesa}}{L_{MQW}}+\frac{\varepsilon_{SiN}A_{cont}}{L_{SiN}}=78.5\text{ pF}$. The RC time constant of the device is then $R_{av}C_{meta}=47.1\text{ nsec}$ and corresponding cut-off frequency is $f_{c}=\left( 2\pi R_{av}C_{meta} \right)^{-1}=3.38 \text{MHz}$.


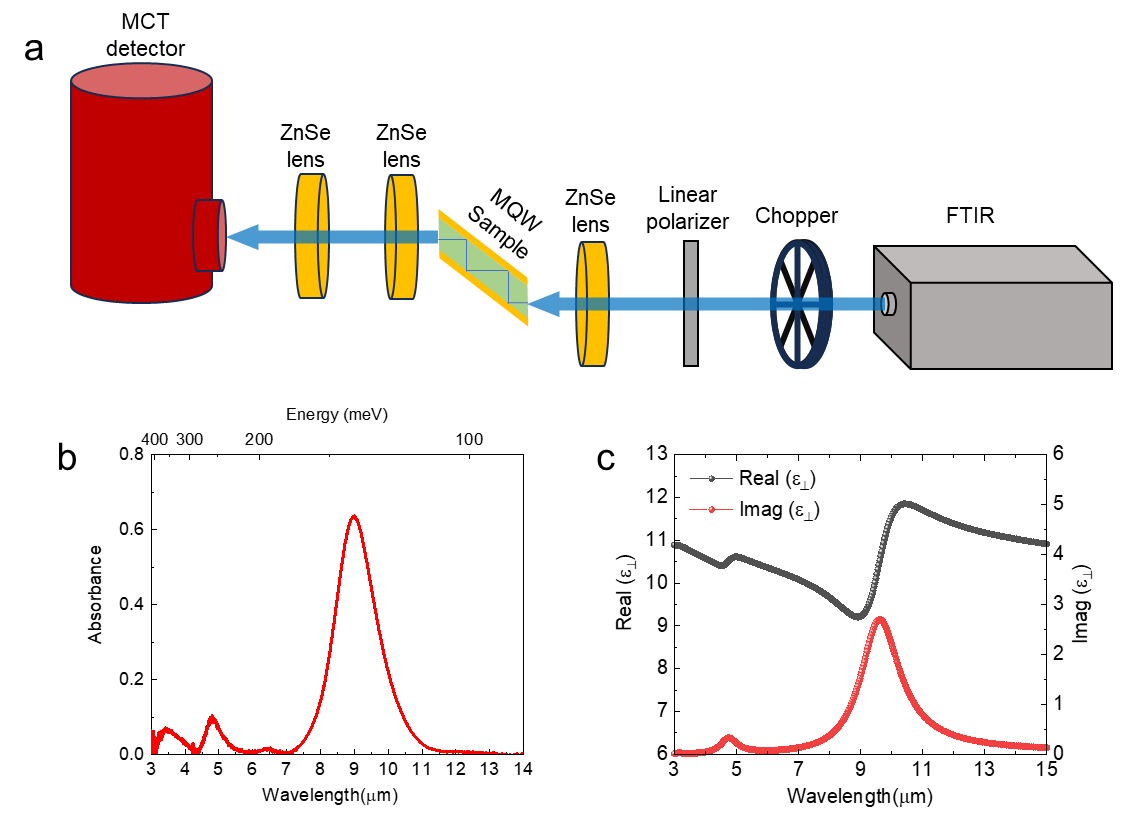


**Supplementary Fig. S1** **a** Optical setup for the intersubband absorption measurement of MQW. **b** Measured Intersubband absorption spectrum by normalizing the TM signal to the TE signal passing through MQW after baseline subtraction processing. **c** Real (black) and imaginary (red) part of the dielectric constant in the surface normal direction as a function of input wavelength.


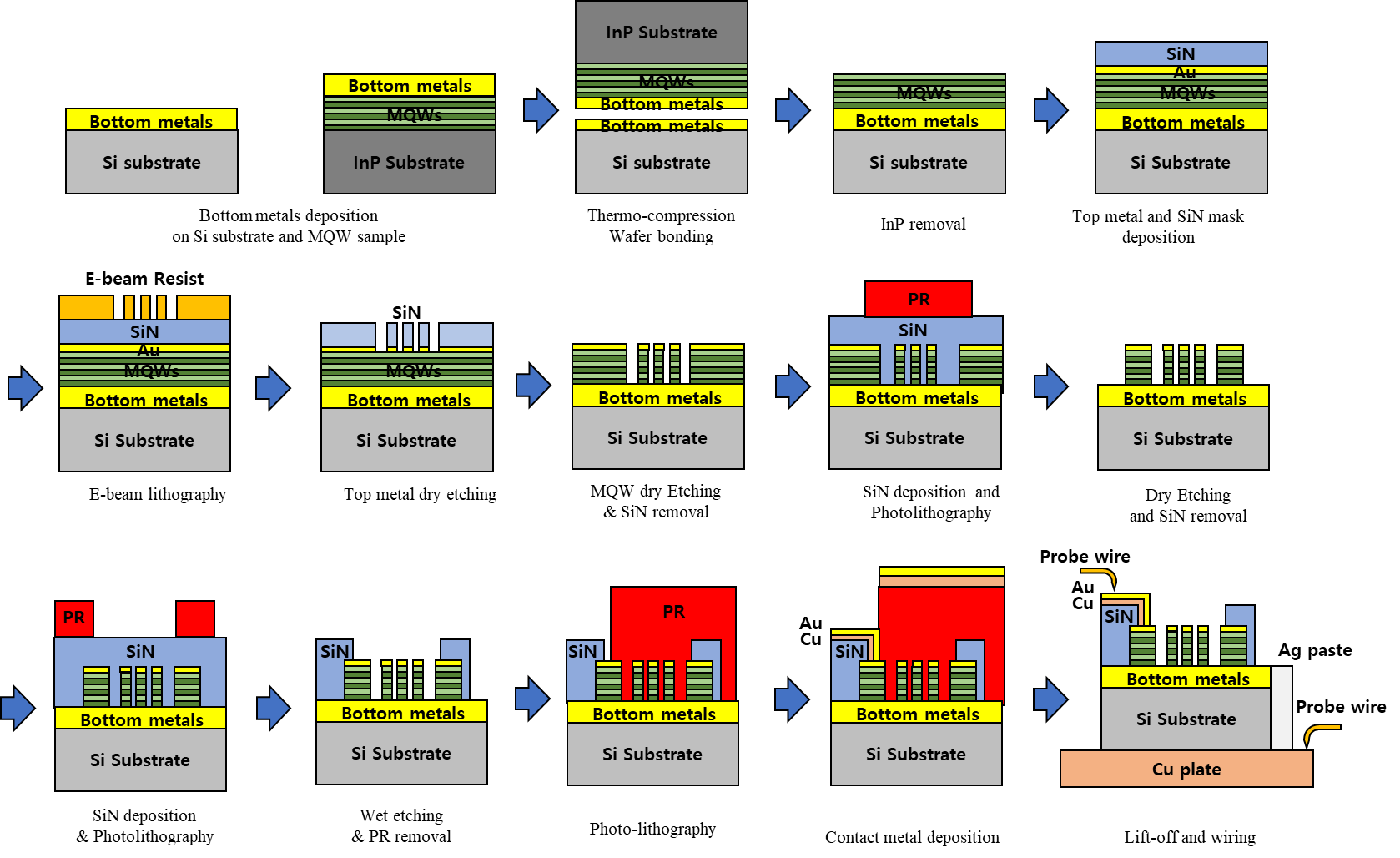


**Supplementary Fig. S2** Metasurface fabrication processes.


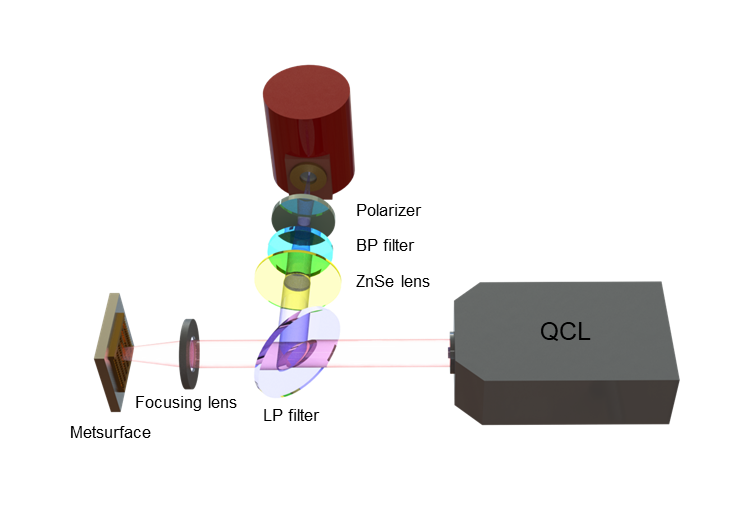


**Supplementary Fig. S3**. Optical setup for the nonlinear optical characterization of the metasurface. The linearly polarized input pump beam from the QCL was directed onto the metasurface, having passed through a long-wavelength pass (LP) filter (with a pass wavelength > 7.2 μm) and a ZnSe aspheric lens (numerical aperture: 0.56, effective focal length: 5 mm). The resulting TH signal traversed the ZnSe lens, passed through the LP filter, and was then focused onto the detector through a linear polarizer and a bandpass (BP) filter (with a pass wavelength of 2.85 – 3.85 μm).


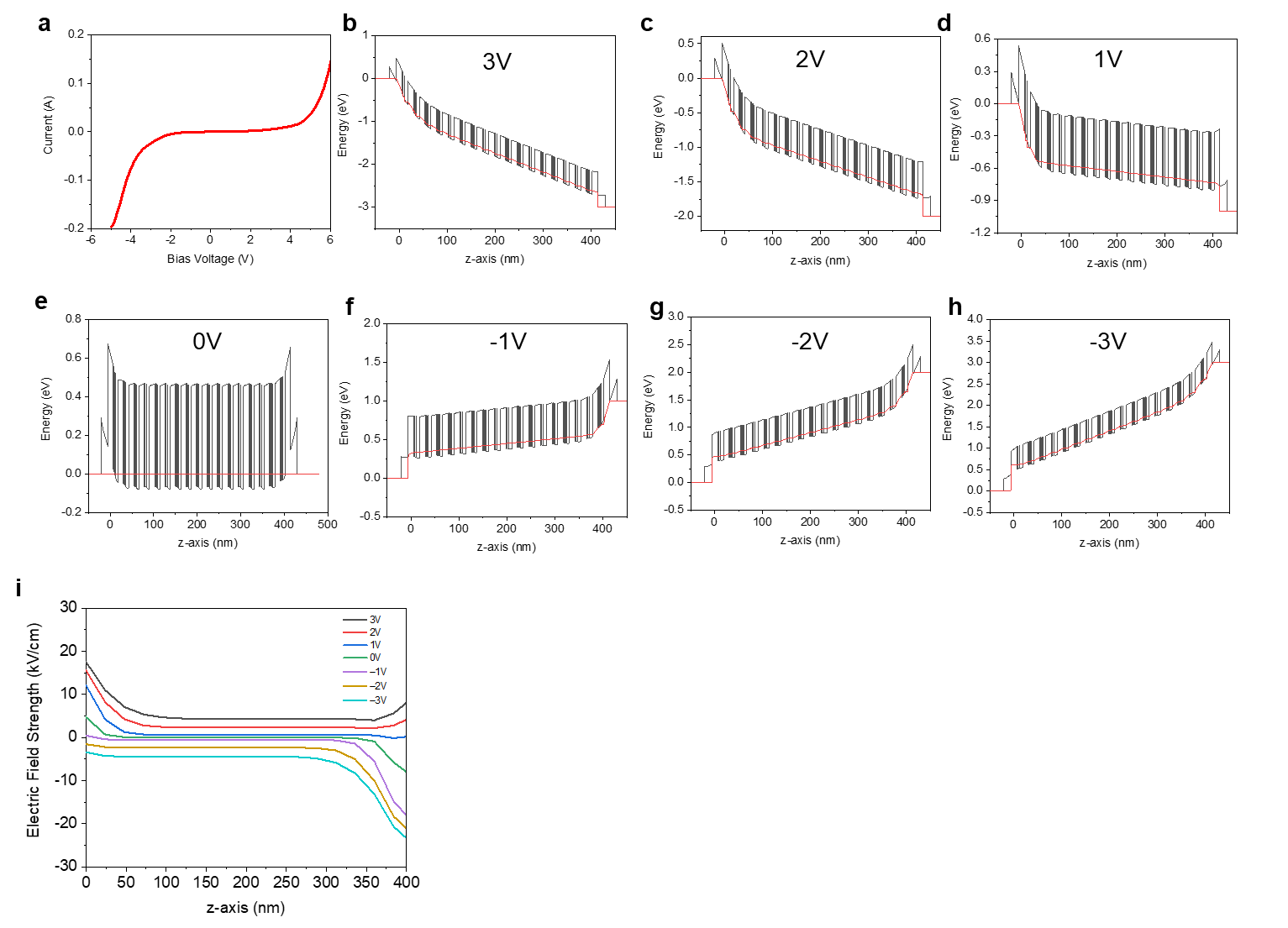


**Supplementary Fig. S4** **a** Current-voltage characteristic of the fabricated metasurface. **b**-**h** Simulation results of the conduction band bending of the MQW and metal layers by applying bias voltages from −3 V to 3 V with a resolution of 1 V, assuming Schottky contact formation. The MQW region ranges from 0 nm to 408 nm with the bottom metallic layer junction at 0 nm and the nanoresonator junction at 414 nm. **i** Electric field strength distribution in the MQW layer over an applied bias voltage range from −3 V to 3 V at a resolution 1 V.


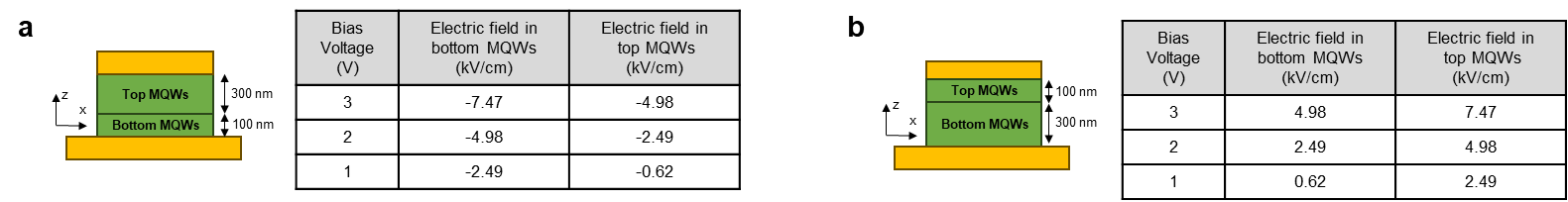


**Supplementary Table S1**. **a**, **b** Table of the calculated averaged electric field strengths for each of the two divided regions of the MQW under (**a)** positive and (**b)** negative bias voltage to the top metallic contact for constructing a MQW model for FDTD simulation. For a positive bias voltage, the thickness of the top MQWs layer is set to 300 nm, while the bottom layer is set to 100 nm. For a negative bias voltage, the top MQWs layer is set to 100 nm, and the bottom layer is set to 300 nm.


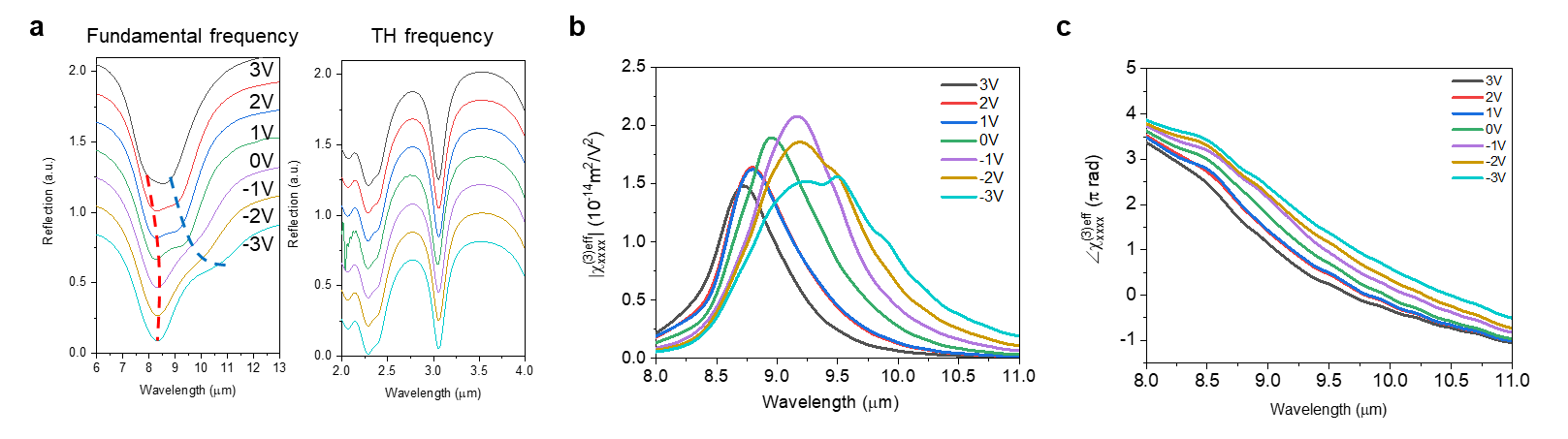


**Supplementary Fig. S5**. **a** Simulated reflection spectra of the metasurface under x-polarized incident light near the (**a**) FF range and (**b**) TH frequency range with consideration of the Schottky contact formation and non-uniform conduction band bending of the MQW layer. **b**, **c** Calculated (**b**) magnitude and (**c**) phase of the effective third-order nonlinear susceptibility of the metasurface.


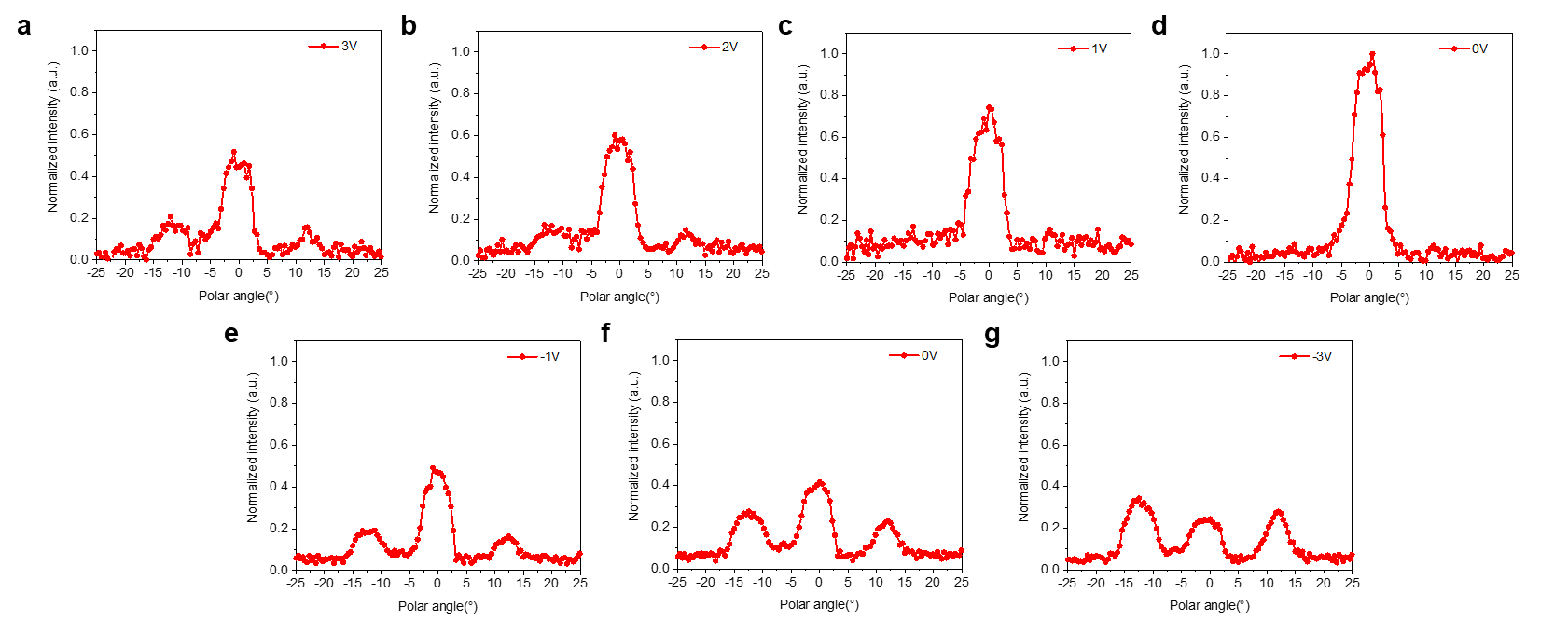


**Supplementary Fig. S6**. **a-g** Measured far-field profile of the THG signal from the phase grating metasurface for applying a single bias to the V_a_ ranging from −3 V to +3 V with a resolution of 1 V and V_b_ remains constant at 0 V. Supercell-period of the metasurface is Γ_2_=14.4 μm.


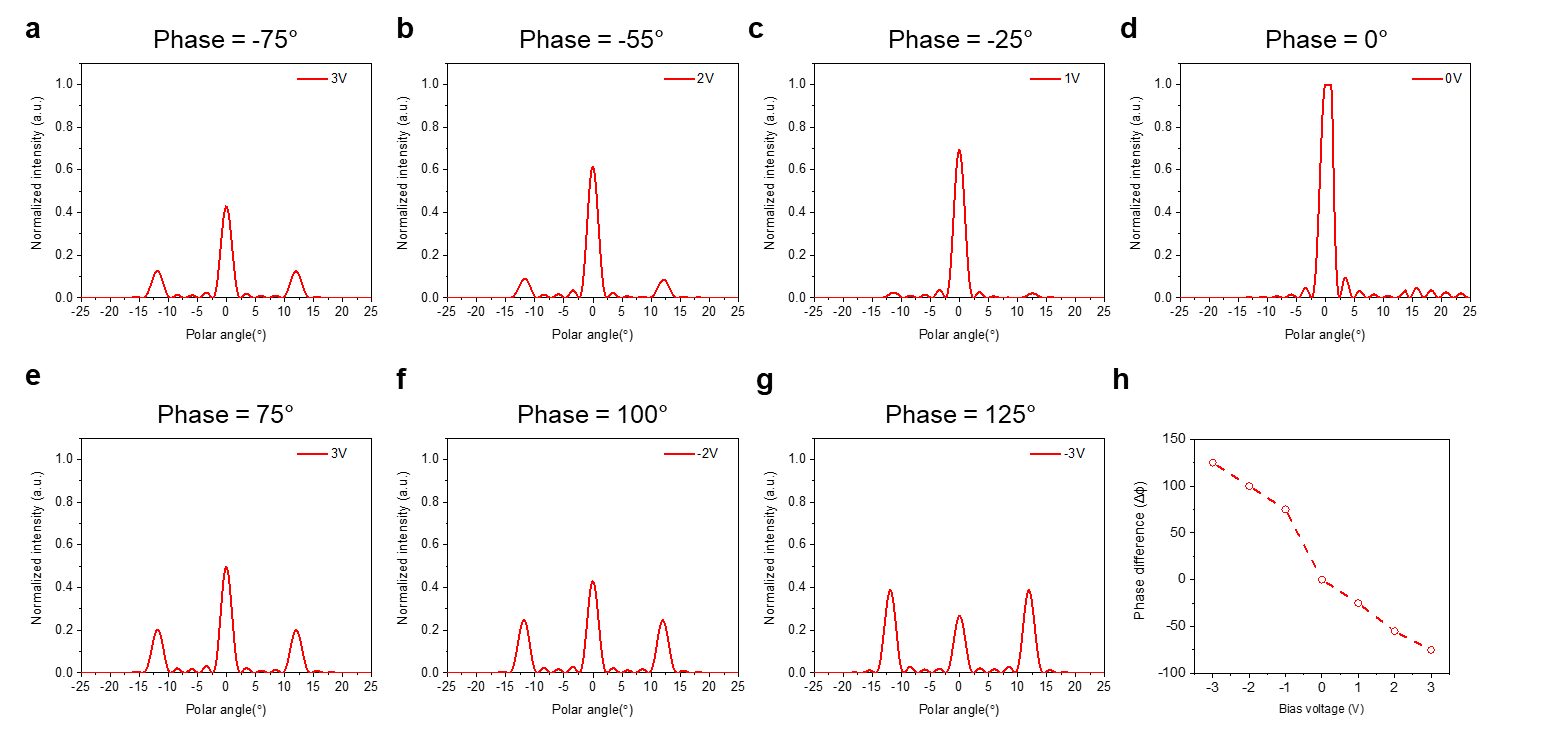


**Supplementary Fig. S7**. **a**-**g** Simulated far-field profile of the THG signal from the phase grating metasurface with a single bias application, where V_a_ from −3 V to +3 V at a 1 V resolution and V_b_ remains constant at 0 V. Supercell-period is Γ_2_=14.4 μm. For the FDTD simulation, dipole antennas are positioned at each unit cell of the metasurface. The amplitude of each dipole antenna is determined by the measured TH power spectrum, and its phase is adjusted based on the bias voltage to align with the measured results. **h** Resultant phases are normalized to the phase according to the bias voltage of 0 V.

**REFERENCES**

S1. Capasso, F., Sirtori, C., and Cho, A. Y. Coupled-quantum-well semiconductors with giant electric-field tunable nonlinear-optical properties in the infrared. *IEEE J. Quantum Electron.* **30**, 1313-1326 (1994).
